# Gold Nanoparticle Inhibits the Tumor-Associated Macrophage M2 Polarization by Inhibiting m^6^A Methylation-Dependent ATG5/Autophagy in Prostate Cancer

**DOI:** 10.1155/ancp/6648632

**Published:** 2025-01-04

**Authors:** Yuanyuan Hao, Feng Duan, Xianning Dong, Ran Bi, Yinzhe Wang, Senqiang Zhu, Jinghai Hu

**Affiliations:** ^1^Department of Urology, The First Hospital of Jilin University, Changchun, China; ^2^Department of Oncology, Qingdao Municipal Hospital, Qingdao, China; ^3^Department of Pathology, The Associated Hospital of Qingdao University, Qingdao, China

**Keywords:** autophagy, gold nanoparticles, m^6^A, prostate cancer

## Abstract

**Background:** This study aims to study how gold nanoparticles (AuNPs) function in the recruitment and polarization of tumor-associated macrophages (TAMs) in hormone-sensitive prostate cancer (HSPC) and castration-resistant prostate cancer (CRPC).

**Methods:** Phorbol ester (PMA)-treated THP-1 cells were cocultured with LNCaP or PC3 cells to simulate TAMs. Macrophage M2 polarization levels were detected using flow cytometry and M2 marker determination. ATG5 expression was detected by western blotting. Luciferase reporter assay was used to analyze the N6-methyladenosine (m^6^A) site activity of ATG5 3′ untranslated regions (3′-UTRs). Methylated RNA immune precipitation (MeRIP)–quantitative polymerase chain reaction (qPCR) was performed to determine the m^6^A levels at ATG5 3′-UTR. Xenograft mouse models were used to determine the function of AuNPs in vivo.

**Results:** Macrophages exhibited reduced M2 polarization in both HSPC and CRPC cells after AuNP treatment which was prevented by induction of autophagy. AuNP treatment decreased the m^6^A levels in the 3′-UTR of ATG5. Mutational analysis of potential m^6^A sites within ATG5 3′-UTR revealed that these sites were required for AuNP regulation, indicating that AuNPs inhibited ATG5 levels in an m^6^A-dependent manner. The mouse model revealed that AuNPs significantly reduced the M2 polarization of TAMs in an autophagy-dependent manner in vivo. This suggests that AuNPs inhibit tumor growth in vivo partially through targeting M2 TAM.

**Conclusion:** The ATG5/autophagy pathway is inhibited by AuNP treatment in an METTL3/m^6^A-dependent manner. AuNPs inhibit the TAM M2 polarization in HSPC and CRPC by inhibiting ATG5/autophagy.

## 1. Introduction

Prostate cancer (PCa) is among the most prevalent malignancies in men globally [1, 2]. The initial growth of PCa requires androgens, suggesting that it should respond well to androgen deprivation therapy [3]. However, in the advanced stages of the disease, hormone-sensitive prostate cancer (HSPC) can become castration-resistant prostate cancer (CRPC) [4]. Currently, no effective treatment is available for CRPC.

Tumor-associated macrophages (TAMs), which differentiate from monocytes, are among the first preinvasive tumor-infiltrating immune cells and persist in invasive cancer [5]. TAMs exist mainly M1- or M2-polarized forms. In the tumor microenvironment, the relationship between M1 and M2 TAMs is crucial for tumor progression. M1 macrophages typically exhibit proinflammatory characteristics. They contribute to tumor cell killing and inhibition. Conversely, M2 macrophages display anti-inflammatory and immunosuppressive traits. They promote tumor growth, invasion, and metastasis and suppress antitumor immunity. Tumor cells and the microenvironment can polarize macrophages toward the M2 phenotype, facilitating immune evasion and tumor progression. An M1 predominance fuels an immunosuppressive environment, aiding tumor survival [6, 7]. The acquisition of protumoral M2 functions by TAMs occurs in both HSPC and CRPC [7]. Therefore, targeting M2 polarization of TAMs has been proposed as a possible strategy for treating HSPC and CRPC.

In recent years, researches have found that specific nanoparticles (NPs) have the potential for antitumor applications [8]. Gold NPs (AuNPs) have diameters ranging from 1 to 100 nm, which can coat various biological macromolecules without affecting their native functions or activities [9]. AuNPs, either alone or in combination with anticancer drugs, exhibit significant cytotoxic effects against various types of cancer cells in both in vitro and in vivo studies [10–14]. AuNPs regulate the polarization switch of TAMs, but their regulatory role remains unclear. Our group recently reported that 60-nm sized AuNPs inhibited the secretion of inflammatory factors, including matrix metalloproteinase-9 (MMP9) and chemokine ligand 2 (CCL2) [10, 11], which are strongly associated with TAM polarization. Therefore, we next further explored the function of 60-nm sized AuNPs on TAM polarization.

Autophagy process could degrade damaged organelles and misfolded proteins [15]. Recent studies suggest that autophagy contributes to the functional mechanisms of macrophages polarization [16, 17]. In this study, we investigated the association between AuNPs and autophagy and the molecular mechanisms regulating M2 polarization of HSPC- and CRPC-derived TAMs.

## 2. Materials and Methods

### 2.1. AuNPs

AuNPs were synthesized as described previously [10, 11]. Briefly, to a boiling solution (95 mL) of HAuCl_4_ (1 mM), 5 mL of disodium citrate (1% w/v) was added dropwise. The solution mixture was stirred continuously for 15 min. The mixture was then allowed to cool down to room temperature taking about 2 h. And the AuNP solution was stored at 4°C away from light for further usage. The characteristics of AuNPs have been reported in detail in our previous publications [10, 11]. The mean size of the AuNPs was 62.2 ± 6 nm [10, 11].

### 2.2. Cell Line Culture

LNCaP (HSPC), PC3 (CRPC), and human monocyte THP-1 cells were cultured in a conventional condition. THP-1 cells were used to establish M0 macrophages by adding 250 ng/mL phorbol ester (PMA), as described previously [18]. For coculturing cancer cells and macrophages, LNCaP or PC3 was seeded on the upper chamber of Corning Costar Transwell inserts (0.4 μm pores; Sigma–Aldrich, St. Louis, MO, USA), which contain membranes permeable to liquids but not cells. The inserts were then placed in compatible wells and plated onto M0 THP-1 cells.

### 2.3. Molecular-Level Detection

Cytokines were measured using enzyme-linked immunosorbent assay (ELISA) kits with purified coatings and detected using biotinylated antibodies. Other proteins were measured by western blotting assay, while mRNA levels were determined by real-time quantitative reverse transcription polymerase chain reaction (RT-qPCR) assay.

### 2.4. Autophagic Vesicle (AV) Counting

The cells were fixed with 25 g/L glutaraldehyde, dehydrated, soaked, embedded, and sliced according to the standard procedure for TEM specimen preparation [19]. The slice thickness was set to 60 nm. Ultrathin slices were stained with a uranium acetate solution for 20 min and treated with a lead solution for 5–6 min. TEM was used to analyze autophagosome and autolysosome formation at different stages. The methods for counting AVs (autophagosomes and autolysosomes) were as described previously [19].

### 2.5. Luciferase Assay

The wild-type (WT) sequence of 3′ untranslated regions (3′-UTRs) of ATG5 and the A-to-T mutant sequence of the specific m^6^A site were synthesized by FitGene (Guangzhou, China). These sequences were inserted into a pLuciferase reporter vector (pLuc). The 293T cells were seeded in 12-well plates at a density of 10^5^ cells/well. Subsequently, cells in designated wells were transfected with 10 ng of the pLuc vector and 1 ng of the control luciferase vector using 0.08 μL of GeneJuice transfection reagent (Merck Millipore, Darmstadt, Germany). After 48 h, luciferase activity was assessed using the Dual-Luciferase Reporter (DLR) Assay (Promega, Madison, WI, USA).

### 2.6. Methylated RNA Immune Precipitation (MeRIP)–qPCR

MeRIP–qPCR analysis was performed as previously described [20]. Briefly, mRNA was fragmented using an RNA fragmentation reagent (Invitrogen, Waltham, MA, USA) and was then immunoprecipitated with anti-m^6^A antibody coupled to Dynabeads (Invitrogen). The m^6^A-containing mRNAs were precipitated with five glycogen (AM9510; Life Technologies, Carlsbad, CA, USA). The m^6^A enrichment was determined using qPCR.

### 2.7. Quantification of Global Methylation

Global m^6^A and m^5^C levels were quantified using EpiQuik m^6^A RNA Methylation Quantification Kit (EpigenTek, Farmingdale, NY, USA) and MethylFlash 5-mC RNA Methylation ELISA Easy Kit (EpigenTek).

### 2.8. Xenograft Mice Model

In vivo experiments were performed in the animal laboratory of Beijing Langke Biotechnology Co., Ltd. (Beijing, China) and were approved by the Institutional Animal Care and Use Committee (IACUC) of the institution (approval number: IACUC-20230111-1). Male severe combined immune deficiency (SCID) mice were propagated in Charles River (MA, USA) and used to construct xenograft models as described previously. Briefly, 6- to 8-week-old male SCID mice were used to establish models of PCa xenografts via surgical orthotopic tumor transplantation. For each mice experiment, at the endpoint, CO_2_ euthanasia was performed on mice gradually until the animals lose consciousness and passed away peacefully.

Approximately 100 μL of the LNCaP cell or PC3 cell suspensions were pulled up into the syringe, and then the syringe was capped with the needle. Subsequently, an amount of 100 μL of Matrigel was drawn up through the needle and was mixed up with the cells by moving the plunger up and down. The mixture (final dose, 5 × 10^6^ cells/mouse) was injected into the prostate capsule until the membrane bulged and separated from the prostate surface. After inoculation, tumor growth and infection were observed daily. The tumor size was monitored weekly. Once the tumor volume reached 100 µL, the animals were categorized into different subgroups according to the treatment method. At the endpoint, blood samples were used to determine cytokine levels, and tumor tissues were collected for the immunofluorescence assay.

Inductively coupled plasma mass spectrometry (ICP-MS) was used to quantify the amount of gold taken up by the tumors, as described previously [8]. Parts of the tumor tissues from the AuNP-treated mice were homogenized and lysed, and the product was used for ICP-MS analysis.

### 2.9. Bone Marrow–Derived Macrophage (BMDM) Cultures and Infusion into the Xenograft Mice

Bone marrow cells were isolated from the mice and inducted into BMDMs as described previously [16]. Subsequently, the cells were stimulated with IL-4 and IL-13 (20 ng/mL) for 48 h to polarize them into M2 BMDM. Next, the cells were harvested and infused into the mice at a concentration of 5 × 10^6^ cells/mouse every second day for 14 days.

### 2.10. Immunofluorescence Assay

The samples were blocked, and the mouse anti-CD68 primary antibodies (Abcam, Cambridge, UK) were applied, followed with CY3-conjugated antimouse IgG (Abcam) treatment. Subsequently, the rabbit anti-CD163 antibodies (Abcam) were introduced, followed with CY5-conjugated antirabbit IgG (Abcam). Immunofluorescence images were captured to identify the regions with double-positive staining.

### 2.11. Statistical Analysis

The results were expressed as means ± SD from at least three separate experiments performed in triplicate. The statistical significance of differences between groups was determined using a two-tailed Student's *t*-test. *p* values less than 0.05 were considered statistically significant. All analyses were performed using SPSS software (IBM Corp., Armonk, NY, USA).

## 3. Results

### 3.1. AuNPs Inhibited M2 Polarization of TAM in Both HSPC and CRPC

**Figure 1:**
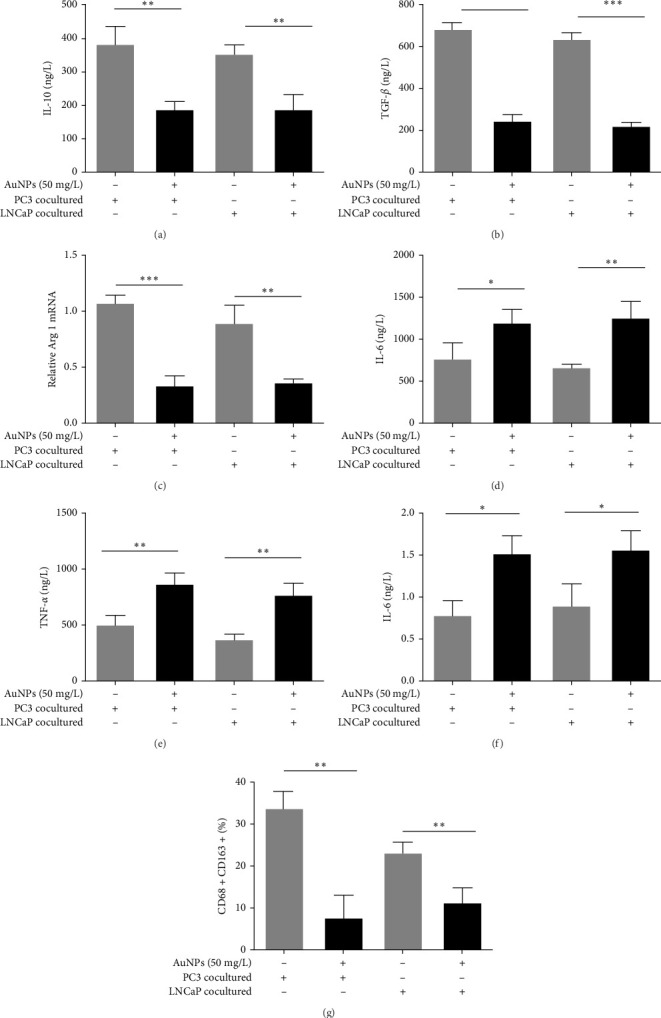
AuNP treatment alters the phenotype of macrophages in response to HSPC and CRPC in vitro. M0 were cocultured with LNCaP and PC3 cells to simulate TAMs, followed by treatment with or without AuNPs at 50 mg/L for 24 h. (A–C) The M2 markers of the macrophages, including (A) IL-10 and (B) TGF-β, which were determined by ELISA on cell-free supernatant, and (C) Arg 1 mRNA levels, which were determined by RT-qPCR on total RNA isolated from the cells, were tested. (D–F) The M1 markers of the macrophages, including (D) IL-6 and (E) TNF-α, which were determined by ELISA on cell-free supernatant, and (F) iNOS mRNA levels, which were determined by RT-qPCR on total RNA isolated from the cells, were tested. (G) The CD163+ (M2 marker) proportion was determined by flow cytometry. The histogram represents the CD163+ proportions. The representative of the flow cytometry diagram in individual group was shown in Figure S1. AuNP, gold nanoparticle; CRPC, castration-resistant prostate cancer; ELISA, enzyme-linked immunosorbent assay; HSPC, hormone-sensitive prostate cancer; RT-qPCR, real-time quantitative reverse transcription polymerase chain reaction; TAMs, tumor-associated macrophages. *⁣*^*∗*^*p* < 0.05, *⁣*^*∗∗*^*p* < 0.01, and *⁣*^*∗∗∗*^*p* < 0.001.

To investigate the role of AuNPs in TAM polarization, M0 macrophages derived from THP-1 cells were cocultured with LNCaP and PC3 cells, with or without 50 mg/L AuNPs, for 24 h to stimulate TAMs. AuNPs decreased the M2 markers, including IL-10, TGF-β, and Arg 1 mRNA (Figure 1A–C) and increased the M1 markers, including IL-6, TNF-α, and iNOS mRNA (Figure 1D–F) in both LNCaP- and PC3-cocultured TAMs. However, the flow cytometry results showed that the proportion of CD163+ (M2 marker) was decreased by AuNPs in both LNCaP- and PC3-cocultured TAMs (Figure 1G, Supporting Information 1: Figure S1). These results indicate that AuNPs inhibited the M2 polarization of TAM in both HSPC and CRPC.

### 3.2. AuNPs Inhibited Autophagy of TAMs in Both HSPC and CRPC

**Figure 2:**
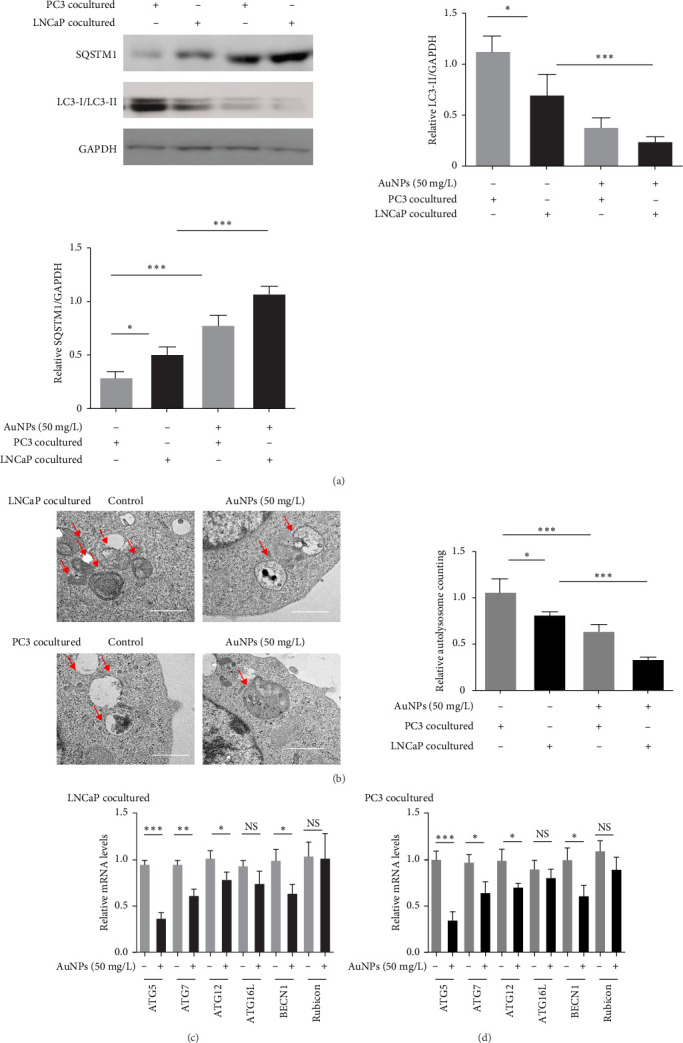
AuNPs inhibited autophagy of M2 TAMs in both HSPC and CRPC. M0 were cocultured with LNCaP and PC3 cells to simulate TAMs, followed by treatment with or without AuNPs at 50 mg/L for 24 h. (A) The protein levels of SQSTM1 and LC3-II/LC3-I were determined by western blotting assay. Left: The representative of the band in individual group. Right: The histogram represents the relative levels of SQSTM1 (SQSTM1/GAPDH ratio) and LC3-II (LC3-II/GAPDH ratio). (B) Observation of autophagy process by TEM. Left: The representative of the TEM version in individual group. Arrows pointing toward typical autophagosomes and autolysosomes. Right: The histogram represents the relative levels of autophagic vesicle counting. (C–D) mRNA levels of several autophagy-related genes (ATGs), which were determined by RT-qPCR on total RNA isolated from the cells, were tested in both (C) LNCaP- and (D) PC3-cocultured TAMs. AuNPs, gold nanoparticles; CRPC, castration-resistant prostate cancer; HSPC, hormone-sensitive prostate cancer; RT-qPCR, real-time quantitative reverse transcription polymerase chain reaction; TAMs, tumor-associated macrophages. *⁣*^*∗*^*p* < 0.05, *⁣*^*∗∗*^*p* < 0.01, and *⁣*^*∗∗∗*^*p* < 0.001.

Autophagy is strongly associated with TAM polarization [21–23]. Therefore, we investigated whether AuNPs alter the autophagy levels in TAMs. First, western blotting results indicated that the LC3-II/GAPDH ratio (autophagy-positive marker) was increased, whereas the SQSTM1/GAPDH ratio (autophagy-negative marker) was decreased in PC3-cocultured TAMs compared with LNCaP-cocultured TAMs. AuNP treatment significantly decreased the LC3-II/GAPDH ratio and increased the SQSTM1/GAPDH ratio (Figure 2A). The number of AVs reflects the degree of autophagy [17]. The number of AVs increased in PC3-cocultured TAMs compared with LNCaP-cocultured TAMs. AuNP treatment significantly decreased the number of AVs in both LNCaP- and PC3-cocultured TAMs (Figure 2B). Furthermore, several autophagy-related genes, including ATG5, ATG7, ATG12, and BECN1, were significantly downregulated by AuNPs, with ATG5 showing the greatest level of inhibition (Figure 2C,D). Therefore, we explored the regulatory mechanisms of AuNPs on ATG5-associated autophagy.

### 3.3. AuNPs Inhibited ATG5 Levels in an m^6^A-Dependent Manner

**Figure 3:**
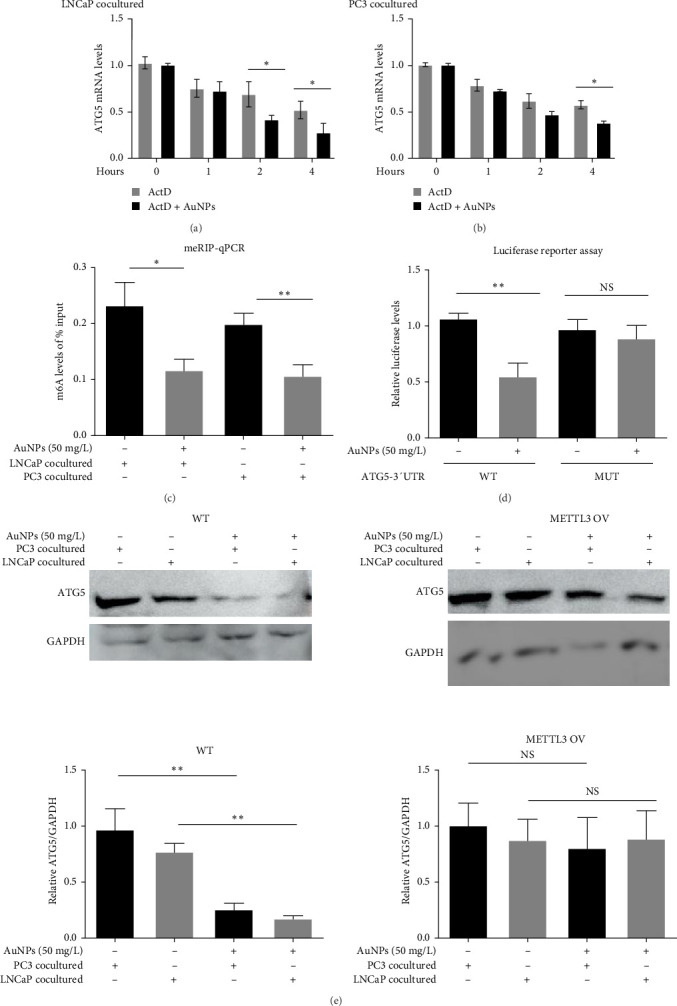
AuNPs inhibited ATG5 levels in an m^6^A-dependent manner. (A–B) M0 macrophages induced from THP-1 were cocultured with LNCaP and PC3 cells to simulate TAMs, together with or without AuNPs at 50 mg/L. Three hours after AuNP treatment, actinomycin D (ActD, transcription inhibitor, 5 mg/L) was added to block transcription, and then the ATG5 mRNA levels were determined in (A) LNCaP-cocultured TAMs and (B) PC3-cocultured TAMs within 4 h after ActD treatment. (C) M0 macrophages induced from THP-1 were cocultured with LNCaP and PC3 cells to simulate TAMs, together with or without AuNPs at 50 mg/L for 24 h. The cells were harvested to perform MeRIP-qPCR assay. (D) Reporter assays using HEK293T cells: The HEK293T cells were transfected with the WT ATG5 3′UTR luciferase reporter or mutant ATG5 3′UTR luciferase reporter for 24 h. Subsequently, these cells were treated with or without AuNPs at 50 mg/L for 24 h; the cells were harvested to perform the luciferase reporter assay. (E) M0 THP-1 cells were treated with METTL3-OV or without METTL3-NC METTL3 overexpressing adenovirus for 48 h and then were cocultured with LNCaP or PC3 for another 24 h to simulate TAMs, together with or without AuNPs at 50 mg/L for 24 h. The ATG5 protein levels were determined by western blotting assay. Left: The representative of the band in individual group. Right: The histogram represents the relative levels of ATG5 (ATG5/GAPDH ratio). 3′-UTR, 3′ untranslated region; AuNPs, gold nanoparticles; MeRIP, methylated RNA immune precipitation; MUT, mutation; qPCR, quantitative polymerase chain reaction; TAMs, tumor-associated macrophages; WT, wild-type. *⁣*^*∗*^*p* < 0.05 and *⁣*^*∗∗*^*p* < 0.01.

mRNA may be induced transcriptionally or post-transcriptionally [24]. AuNPs regulate some key targets through post-transcriptional regulation [25]. Therefore, we investigated whether AuNPs regulate ATG5 levels post-transcriptionally. In AuNP-treated LNCaP- and PC3-cocultured TAMs, ATG5 mRNA decayed significantly under actinomycin D (ActD, transcription inhibitor, 5 mg/L) treatment, and AuNPs significantly accelerated this decay (Figure 3A,B), confirming their post-transcriptional regulatory effects on ATG5.

The m^6^A methylation in 3′-UTR is a recently discovered mechanism of post-transcriptional regulation of RNA stability [26, 27]. Next, we focused on the m^6^A methylation-dependent regulation of ATG5 by AuNPs for the following reasons: (1) It has been reported that AuNP treatment can affect DNA methylation [28]; (2) we found that AuNP treatment could decrease global m^6^A methylation levels but had little impact on global m^5^C methylation levels (Supporting Information 2: Figure S2); and (3) we found that METTL3, which is considered to be the most important m^6^A writer [26, 27], was downregulated by AuNP treatment (Supporting Information 3: Figure S3).

The MeRIP–qPCR results revealed that ATG5 mRNA had lower m^6^A levels following AuNP treatment, confirming our hypothesis that AuNPs can regulate m^6^A methylation-dependent modification of ATG5 mRNA (Figure 3C). We constructed a luciferase reporter containing WT ATG5 3′-UTR or the ATG5 3′-UTR with specific m^6^A site mutations (MUTs), as described previously [20], and transiently transfected them into HEK293T cells. The luciferase level of WT group of ATG5 3′-UTR was significantly decreased in response to AuNPs, whereas it was not affected by AuNPs in the MUT group of ATG5 3′-UTR (Figure 3D), indicating that m^6^A regulation was the main cause of AuNP downregulation of ATG5 RNA stability.

Next, we added METTL3 overexpression (METTL3-OV group) adenovirus or negative control (METTL3-NC group) adenovirus to M0 THP-1 cells and incubated them for 48 h. ATG5/GAPDH and LC3-II/GAPDH were both significantly enhanced in the METTL3-OV group compared with the METTL3-NC group, confirming that METTL3 enhances ATG5/autophagy levels (Supporting Information 4: Figure S4). Subsequently, M0 macrophages in the METTL3-NC and METTL3-OV groups were cocultured with LNCaP and PC3 cells, with or without 50 mg/L AuNPs, for 24 h to stimulate TAMs. The protein levels of ATG5 were decreased by AuNPs in the METTL3-NC group, and this effect was reversed in the METTL3-OV group (Figure 3E). Collectively, these results demonstrate that AuNPs inhibited ATG5 levels in an METTL3/m^6^A-dependent manner.

### 3.4. ATG5-Associated Autophagy Is Responsible for Mediating the AuNP Effect on TAM M2 Polarization

Next, we analyzed the role of ATG5-associated autophagy in AuNP-mediated M2 polarization. We added ATG5 overexpression (ATG5-OV group) adenovirus or negative control (ATG5-NC group) adenovirus to M0 THP-1 cells for 48 h. Western blotting results showed that LC3-II/GAPDH increased significantly in the ATG5-OV group compared with the ATG5-NC group (Supporting Information 5: Figure S5).

**Figure 4:**
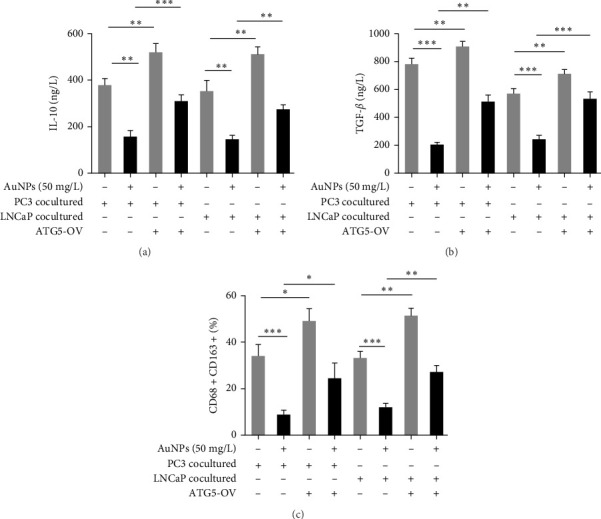
ATG5-associated autophagy mediates AuNP effect on M2 TAMs in both HSPC and CRPC. M0 THP-1 cells were treated with ATG5-OV or without ATG5-NC ATG5 overexpressing adenovirus for 48 h and then were cocultured with LNCaP or PC3 for another 24 h to simulate TAMs, together with or without AuNPs at 50 mg/L for 24 h. (A–B) The M2 markers of the macrophages, including (A) IL-10 and (B) TGF-β, which were determined by ELISA on cell-free supernatant, were tested. (C) The CD163+ (M2 marker) proportion was determined by flow cytometry. The histogram represents the CD163+ proportions. The representative of the flow cytometry diagram in individual group was shown in Figure S1. AuNP, gold nanoparticle; CRPC, castration-resistant prostate cancer; ELISA, enzyme-linked immunosorbent assay; HSPC, hormone-sensitive prostate cancer; TAMs, tumor-associated macrophages. *⁣*^*∗*^*p* < 0.05, *⁣*^*∗∗*^*p* < 0.01, and *⁣*^*∗∗∗*^*p* < 0.001.

Subsequently, M0 THP-1 cells in the ATG5-NC and ATG5-OV groups were cocultured with LNCaP and PC3 cells, with or without 50 mg/L AuNPs for 24 h to simulate TAMs. ATG5-OV enhanced the IL-10 (Figure 4A), TGF-β (Figure 4B), and CD163+ proportions, confirming that ATG5/autophagy is the positive regulatory factor for TAM M2 polarization. AuNPs decreased the IL-10 (Figure 4A), TGF-β (Figure 4B), and CD163+ proportions (Figure 4C) in both LNCaP and PC3 cocultured macrophages, and ATG5-OV partially reversed this downregulation compared with the ATG5-NC group (Figure 4A–C). These results indicate that ATG5-associated autophagy is at least partially responsible for mediating the effect of AuNPs on M2 polarization of TAMs.

### 3.5. AuNPs Regulated M2 Polarization in an Autophagy-Dependent Manner in Vivo

**Figure 5:**
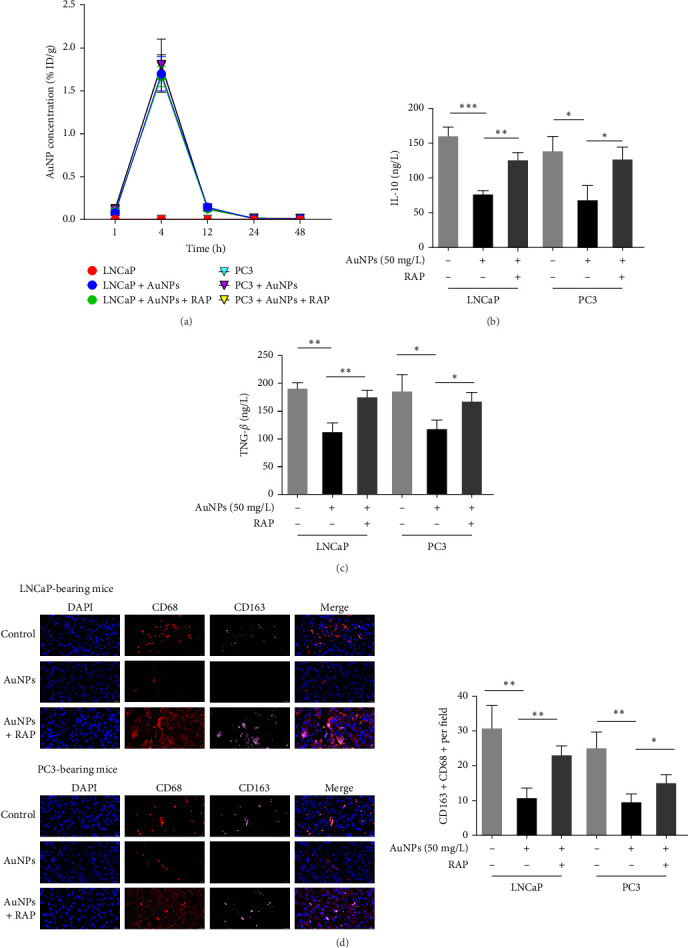
AuNPs regulated M2 polarization in an autophagy-dependent manner in vivo. AuNPs/RAP-treated xenograft mouse models were constructed using LNCaP or PC3 according to the “Materials and Methods” section. Once the tumor volume reached 100 mm^3^, the mice were categorized into subgroups and treated with 1.0 mg kg^−1^ AuNPs (AuNPs group, *n* = 8), 1.0 mg kg^−1^ AuNPs plus 5.0 mg kg^−1^ rapamycin (AuNPs + RAP group, *n* = 8, rapamycin is autophagy inducer), or an equal amount of saline (NC group, *n* = 8) intravenously every other day for 14 days. (A) First dosing cycle (48 h), tumor AuNP concentration after AuNP treatment in xenograft mouse models. (B–C) At the endpoint, the blood samples were used to determine the (B) IL-10 and (C) TGF-β in each group. (D) At the endpoint, immunofluorescence assays were performed. M2-like macrophages were analyzed on the basis of nuclei (blue), CD68 (orange, primarily in cytoplasm), and CD163 (red, cell surface marker). Left: The representative of the images are shown of tissues from the mice. Right: The histogram represents the CD163+CD68+ counting per field. AuNPs, gold nanoparticles. *⁣*^*∗*^*p* < 0.05, *⁣*^*∗∗*^*p* < 0.01, and *⁣*^*∗∗∗*^*p* < 0.001.

Next, we analyzed the role of autophagy in AuNP-regulated M2 polarization in vivo using a AuNPs treated murine xenograft model. The concentration of AuNPs in tumor tissues every treatment cycle (every 48 h) was shown in Figure 5A. There were no significant differences in the concentrations of AuNPs among the different treatment groups (Figure 5A).

At the endpoint on day 14, we found that AuNP treatment decreased the M2 markers, IL-10, and TGF-β levels in blood cytokines of both LNCaP and PC3 xenograft mice, whereas rapamycin cotreatment reversed this effect (Figure 5B,C).

Next, we used double immunofluorescence staining to detect the number of M2 macrophages. There were fewer CD68+ CD163+ cells (M2 macrophages) in the AuNP-treated mice than in the control group, and this function was reversed in the AuNP + RAP group (Figure 5D). Collectively, these results indicate that AuNPs regulate M2 polarization in an autophagy-dependent manner in vivo.

### 3.6. AuNPs Inhibit Tumor Growth In Vivo At Least Partially Through Targeting M2 TAM

**Figure 6:**
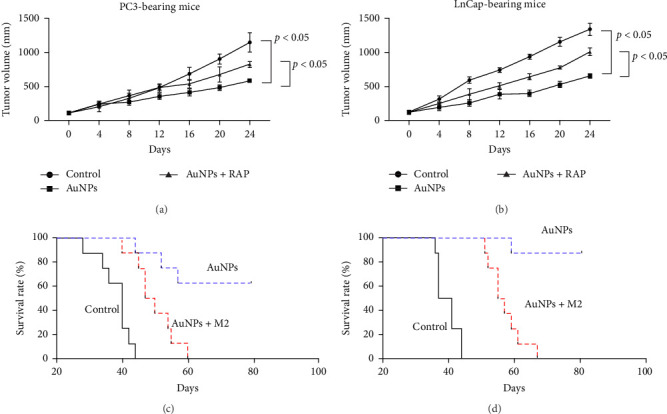
AuNPs inhibit tumor growth in vivo at least partially through targeting M2 TAM. AuNPs/M2 macrophage-treated xenograft mouse models were constructed using LNCaP or PC3 according to the “Materials and Methods” section. Once the tumor volume reached 100 mm^3^, the mice were categorized into subgroups and treated with 1.0 mg kg^−1^ AuNPs (AuNPs group, *n* = 8), 1.0 mg kg^−1^ AuNPs plus M2 BMDM infusion (AuNPs + M2 group, *n* = 8), or an equal amount of saline (NC group, *n* = 8) intravenously every other day for 14 days. (A–B) Tumor volumes were measured every 4 days after AuNP treatment in (A) PC3-bearing mice and (B) LNCaP-bearing mice. (C–D) The survival time of mice was subsequently observed within 80 days, without any additional treatment, in (C) PC3-bearing mice and (D) LNCaP-bearing mice. AuNPs, gold nanoparticles; BMDM, bone marrow–derived macrophages; TAM, tumor-associated macrophage.

Next, we explored the role of AuNP-regulated M2 TAM on tumor growth in vivo. AuNPs significantly decreased the tumor volume compared with that in the NC group. The therapeutic effects of AuNPs were reversed in the AuNP + M2 group (Figure 6A,B). The survival time of mice was subsequently observed within 80 days, without any additional treatment, and we found that the 2-week treatment with AuNPs significantly increased the overall survival rate, while this was reversed in the AuNP + M2 group (Figure 6C,D). These results indicate that AuNPs inhibit tumor growth in vivo, at least partially through targeting M2 TAM.

## 4. Discussion

AuNPs possess unique properties that facilitate their therapeutic application in cancer treatment. The antitumor functions of AuNPs can be roughly divided into three categories. First, AuNPs can be functionalized with drugs or therapeutic agents and used as carriers for targeted drug delivery to tumor cells [29]. Their surface properties and size can be tailored to enhance the drug loading and release efficiency. Second, AuNPs convert near-infrared light into heat, selectively killing tumor cells through hyperthermia. This approach has been used for efficient tumor ablation with minimal damage to healthy tissues [30]. Third, AuNPs are strongly toxic to tumor cells, both in vivo and in vitro [8, 10]. Several recent studies have shown that AuNPs enhance the efficacy of immunotherapy against various types of cancer by modulating the immune response. Dey et al. [31] reported that dendritic cells and TAMs are targeted by AuNPs. Taratummarat et al. [32] demonstrated that AuNPs regulate M2 macrophage polarization. M2 macrophages are immunosuppressive and protumorigenic and thus play an important role in tumor progression and metastasis [5]. In this study, we found that AuNPs decreased the production of TGF-β and IL-10, the M2 markers of TAMs in both HSPC and CRPC, and downregulated the proportion of CD163+ macrophages. These results suggest that AuNPs exert their antitumor activity by regulating the polarization and aggregation of TAMs. Furthermore, we confirmed that the regulatory effect of AuNPs on macrophage function was due to direct autophagy.

The relationship between autophagy and macrophage M2 polarization is complex. Chang et al. [33] reported that autophagy can promote M2 polarization via a ferroptosis-dependent pathway. Sanjurjo et al. [34] found that the upregulation of ID3 by autophagy was the main factor contributing to M2 polarization. However, other studies have shown that autophagy also inhibits macrophage M2 polarization under certain conditions [35]. This indicates that autophagy may have a dual effect on TAM function. In our research model, we found that AuNPs downregulated the expression of ATG5, leading to a decrease in autophagy, thereby inhibiting M2 polarization and macrophage recruitment.

Using ActD, we found that AuNPs exert post-transcriptional regulation of ATG5 expression. A comprehensive analysis revealed that AuNPs regulate the m^6^A level of the ATG5 3′-UTR. Previous studies indicate that the m^6^A modification of the 3′-UTR of ATG5 mRNA contributes to its stability, leading to altered ATG5 expression and subsequent effect on autophagy [20]. The formation of m^6^A is triggered by m^6^A “writers,” including METTL3, METTL14, and WTAP [26, 27]. Our results demonstrate that METTL3 overexpression abrogates the inhibitory effect of AuNPs on ATG5, indicating that the effect of AuNPs on ATG5 is mediated via METTL3-dependent m^6^A modification.

Further animal experiments are required to validate these cytology-based experimental results. The internalization of AuNPs by tumor cells or TAMs in animals differs greatly from the results of cytology studies, and the efficiency of AuNPs in vivo differs from that in vitro [36]. However, the toxic effects of AuNPs on normal histiocytes restrict the dosage of AuNPs that can be used in animal studies [37]. Therefore, it is necessary to determine a reasonable dosage of AuNPs with high activity and low toxicity based on animal experiments. Over the past several years, significant advances have been made in research investigating the use of AuNPs to regulate tumor growth using animal models [38, 39]. In our previous study, using xenograft mice, we found that AuNPs at a dose of 1.0 mg kg^−1^ inhibited tumor growth and prolonged the survival time of mice [10, 11]. In this study, we found that the number of M2 TAMs decreased significantly after treatment with AuNPs at this concentration. Furthermore, the regulatory role of AuNPs against M2 TAMs was significantly reversed when combined with RAP treatment in vivo, which establishes the key role of the ATG5/autophagy pathway in regulating M2 TAMs in vivo.

The presence of M2 macrophages is closely associated with tumor invasion, metastasis, and treatment resistance [40]. Interfering with its activity may alter the trajectory of tumor development, enhance the immune response, and improve treatment efficacy [40]. Therefore, understanding the mechanism of action of M2 macrophages in tumors is crucial for the development of new antitumor treatment strategies. In this study, we confirmed that AuNPs inhibit PCa cell growth in vivo, at least partially, by targeting M2 TAMs. We have previously demonstrated that AuNPs can directly target tumor cells in vivo. Collectively, these results demonstrate that the antitumor effect of AuNPs is a comprehensive process that targets multiple cells. These findings provide a novel direction for future PCa therapy.

## Data Availability

The data that support the findings of this study are available from the corresponding author upon reasonable request.

## Supporting Information

Supporting Information 1 Figure S1: Related to Figure 1G and Figure 4C, the CD163+ (M2 marker) proportion was determined by flow cytometry. Representative scatter plot in individual group was shown in this figure.

Supporting Information 2 Figure S2: AuNPs decreased global m6A levels of M2 TAMs in both HSPC and CRPC. M0 were cocultured with LNCaP and PC3 cells to simulate TAMs, followed by treatment with or without AuNPs at 50 mg/L for 24 h. Global m6A levels and global m5C levels were determined.

Supporting Information 3 Figure S3: AuNPs decreased METTL3 protein levels of M2 TAMs in both HSPC and CRPC. M0 were cocultured with LNCaP and PC3 cells to simulate TAMs, followed by treatment with or without AuNPs at 50 mg/L for 24 h. The protein levels of METTL3 were determined by western blotting assay. Top: The representative of the band in individual group. Bottom: The histogram represents the relative levels of METTL3 (METTL3/GAPDH ratio).

Supporting Information 4 Figure S4: METTL3 overexpressed macrophage construction. M0 macrophages were treated with METTL3 overexpression (METTL3-OV group) adenovirus or negative control (METTL3-NC group) adenovirus for 48 h. The protein levels of METTL3, ATG5, and LC3-I/II protein levels were determined by western blotting assay. Top: The representative of the band in individual group. Bottom: The histogram represents the relative levels of METTL3, ATG5, and LC3-I/II (METTL3/GAPDH ratio, ATG5/GAPDH ratio, and LC3-II/GAPDH ratio).

Supporting Information 5 Figure S5: ATG5 overexpressed macrophage construction. M0 macrophages were treated with ATG5 overexpression (ATG5-OV group) adenovirus or negative control (ATG5-NC group) adenovirus for 48 h. The protein levels of ATG5 and LC3-I/II protein levels were determined by western blotting assay. Top: The representative of the band in individual group. Bottom: The histogram represents the relative levels of ATG5 and LC3-I/LC3-II (ATG5/GAPDH ratio and LC3-II/GAPDH ratio).
