# Supplementary figures and images for: Gold Nanoparticle Inhibits the Tumor-Associated Macrophage M2 Polarization by Inhibiting m6A Methylation-Dependent ATG5/Autophagy in Prostate Cancer
Source: Anal Cell Pathol (Amst). 2025 Jan 4;2025:6648632. doi: 10.1155/ancp/6648632 (PMC11724730; doi:10.1155/ancp/6648632)

AuNPs (50 mg/L)  
ATG5-OV

-

+

+

-

-

+

PC3 cocultured

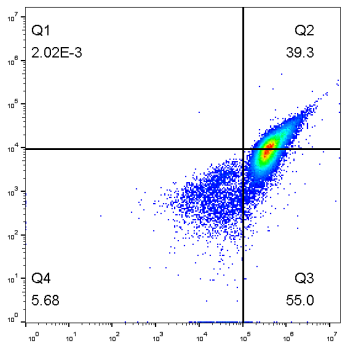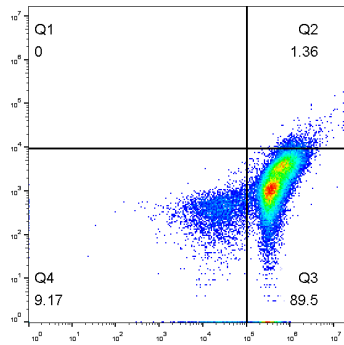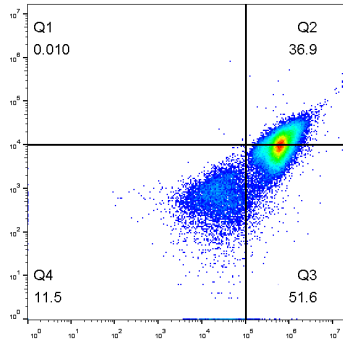

LnCaP cocultured

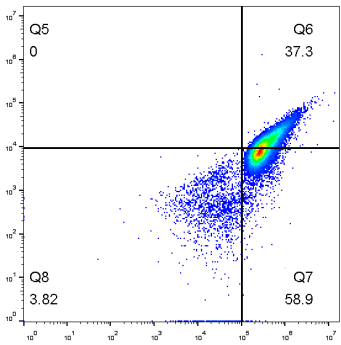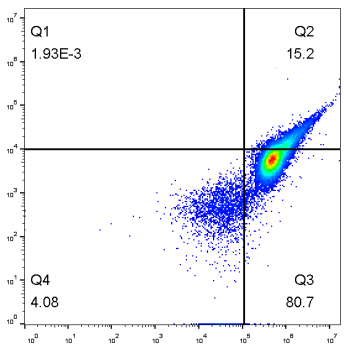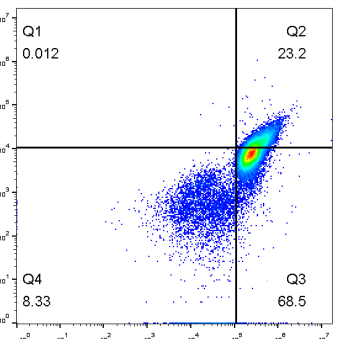

CD163

CD68

Supplement: Supporting Information 1 — Figure S1: Related to Figure 1G and Figure 4C, the CD163+ (M2 marker) proportion was determined by flow cytometry. Representative scatter plot in individual group was shown in this figure. [file 6648632.f1.pdf]

**A**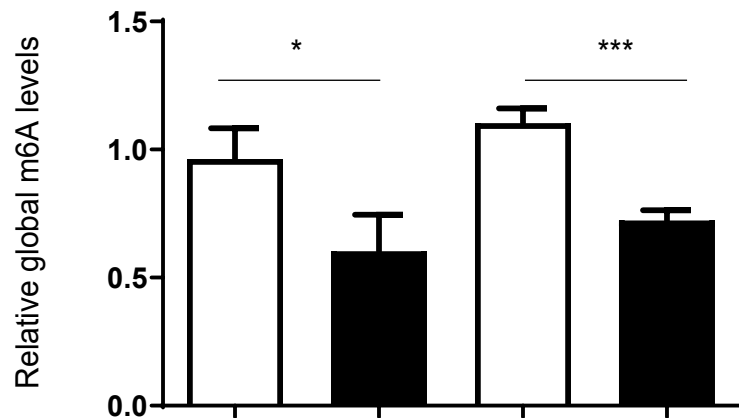

|                  |   |   |   |   |
|------------------|---|---|---|---|
| AuNPs (50 mg/L)  | - | + | - | + |
| PC3 cocultured   | + | + | - | - |
| LnCaP cocultured | - | - | + | + |

**B**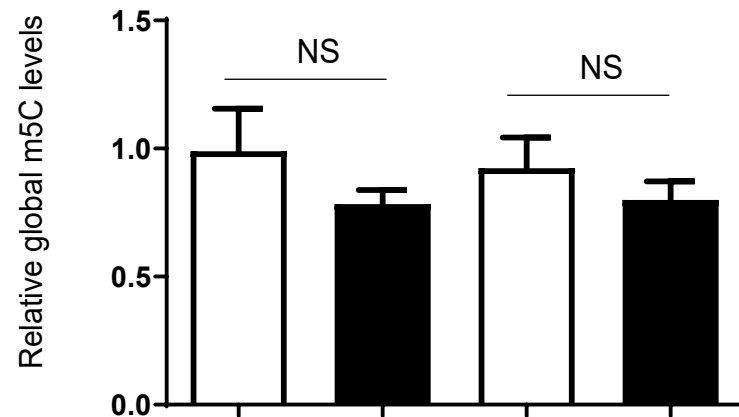

|                  |   |   |   |   |
|------------------|---|---|---|---|
| AuNPs (50 mg/L)  | - | + | - | + |
| PC3 cocultured   | + | + | - | - |
| LnCaP cocultured | - | - | + | + |

Supplement: Supporting Information 2 — Figure S2: AuNPs decreased global m6A levels of M2 TAMs in both HSPC and CRPC. M0 were cocultured with LNCaP and PC3 cells to simulate TAMs, followed by treatment with or without AuNPs at 50 mg/L for 24 h. Global m6A levels and global m5C levels were determined. [file 6648632.f2.pdf]

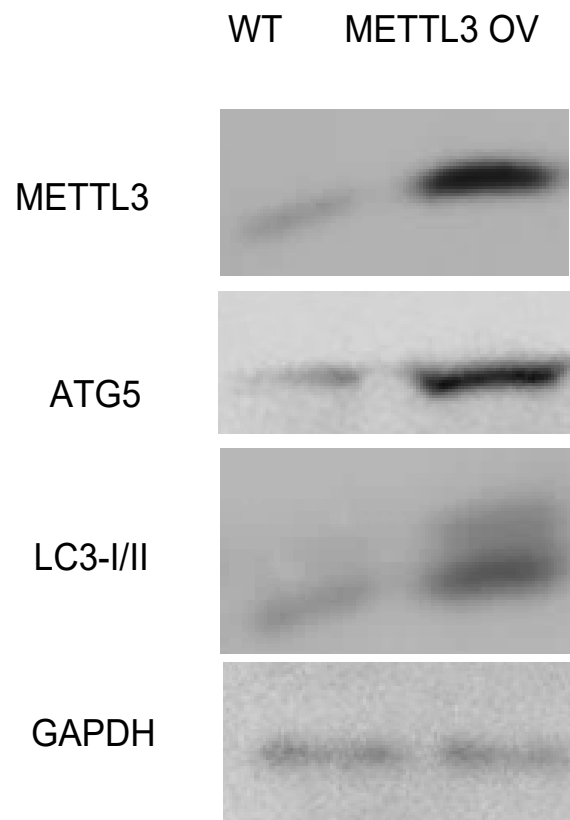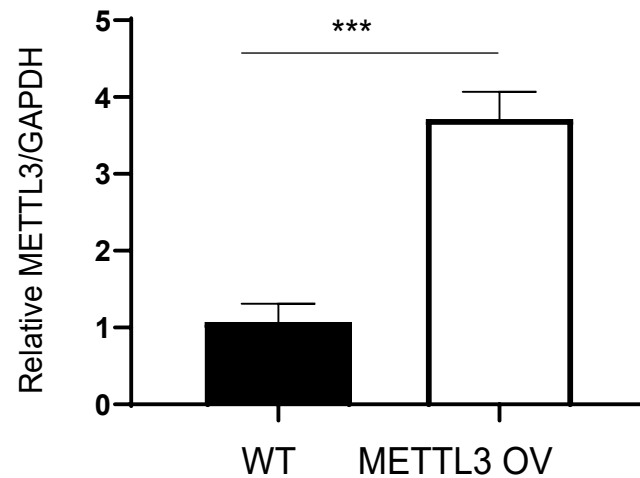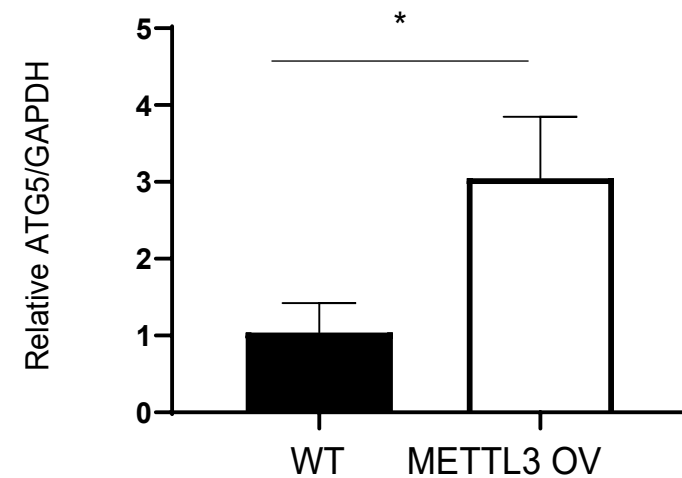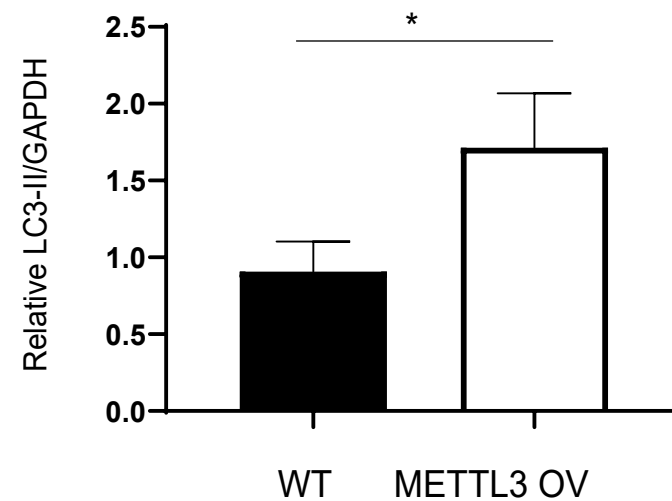

Supplement: Supporting Information 4 — Figure S4: METTL3 overexpressed macrophage construction. M0 macrophages were treated with METTL3 overexpression (METTL3-OV group) adenovirus or negative control (METTL3-NC group) adenovirus for 48 h. The protein levels of METTL3, ATG5, and LC3-I/II protein levels were determined by western blotting assay. Top: The representative of the band in individual group. Bottom: The histogram represents the relative levels of METTL3, ATG5, and LC3-I/II (METTL3/GAPDH ratio, ATG5/GAPDH ratio, and LC3-II/GAPDH ratio). [file 6648632.f4.pdf]

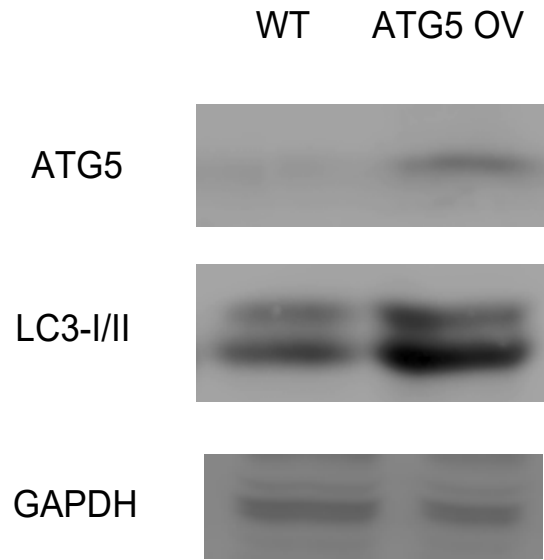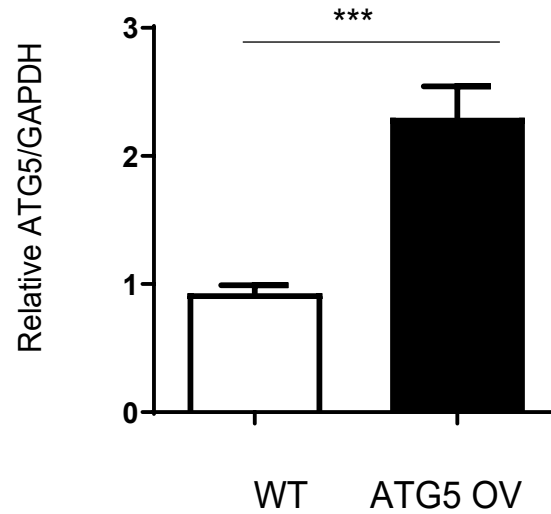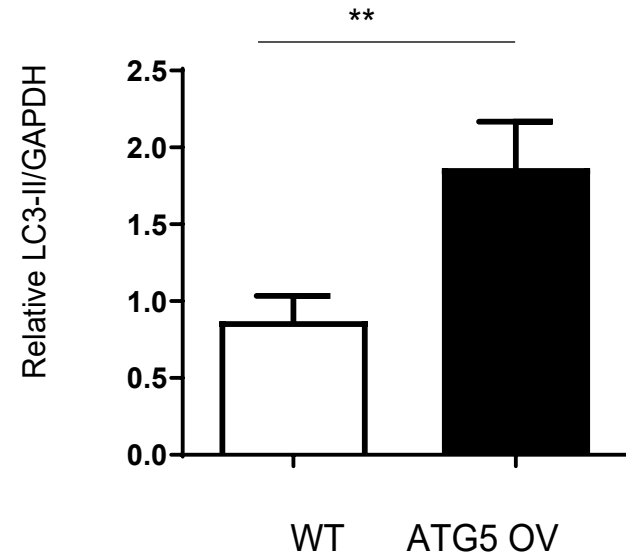

Supplement: Supporting Information 5 — Figure S5: ATG5 overexpressed macrophage construction. M0 macrophages were treated with ATG5 overexpression (ATG5-OV group) adenovirus or negative control (ATG5-NC group) adenovirus for 48 h. The protein levels of ATG5 and LC3-I/II protein levels were determined by western blotting assay. Top: The representative of the band in individual group. Bottom: The histogram represents the relative levels of ATG5 and LC3-I/LC3-II (ATG5/GAPDH ratio and LC3-II/GAPDH ratio). [file 6648632.f5.pdf]
