# Supplementary material for: Gold Nanoparticle Inhibits the Tumor-Associated Macrophage M2 Polarization by Inhibiting m6A Methylation-Dependent ATG5/Autophagy in Prostate Cancer
Source: Anal Cell Pathol (Amst). 2025 Jan 4;2025:6648632. doi: 10.1155/ancp/6648632 (PMC11724730; doi:10.1155/ancp/6648632)
Supplement: Supporting Information 3 — Figure S3: AuNPs decreased METTL3 protein levels of M2 TAMs in both HSPC and CRPC. M0 were cocultured with LNCaP and PC3 cells to simulate TAMs, followed by treatment with or without AuNPs at 50 mg/L for 24 h. The protein levels of METTL3 were determined by western blotting assay. Top: The representative of the band in individual group. Bottom: The histogram represents the relative levels of METTL3 (METTL3/GAPDH ratio). [file 6648632.f3.pdf]

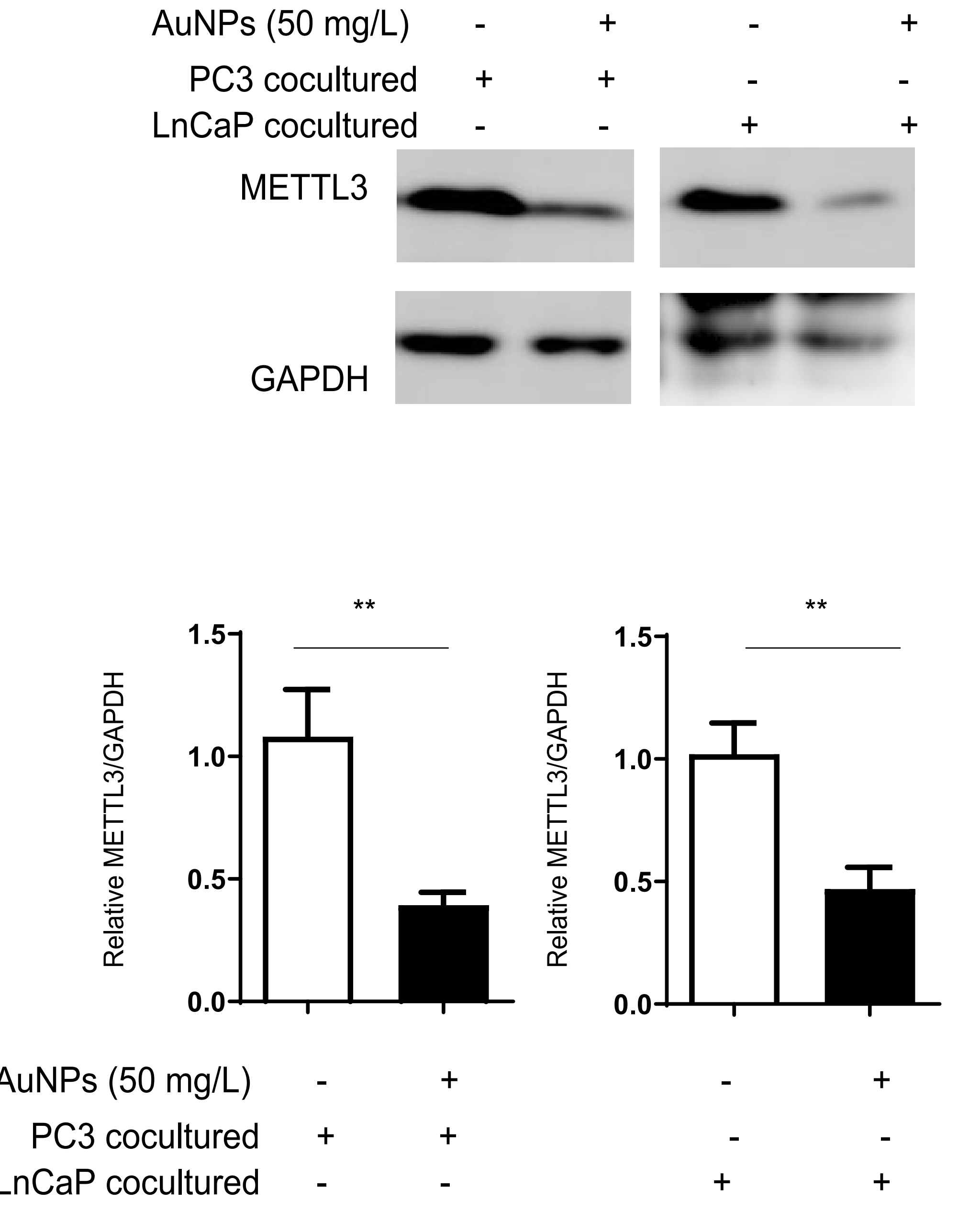
AuNPs (50 mg/L) - + - +
PC3 cocultured + + - -
LnCaP cocultured - - + +
METTL3
GAPDH
**
**
Relative METTL3/GAPDH
Relative METTL3/GAPDH
1.5
1.0
0.5
0.0
AuNPs (50 mg/L) - + - +
PC3 cocultured + + - -
LnCaP cocultured - - + +
